# Inhibition of the Deep and Intermediate Layers of the Superior Colliculus Disrupts Sensorimotor Gating in Monkeys

**DOI:** 10.3389/fnbeh.2020.610702

**Published:** 2020-12-22

**Authors:** Hannah F. Waguespack, Brittany L. Aguilar, Ludise Malkova, Patrick A. Forcelli

**Affiliations:** ^1^Interdisciplinary Program in Neuroscience, Georgetown University, Washington, DC, United States; ^2^Department of Pharmacology & Physiology, Georgetown University, Washington, DC, United States; ^3^Department of Neuroscience, Georgetown University, Washington, DC, United States

**Keywords:** macaque, superior collicullus, acoustic startle reflex, pre-pulse inhibition (PPI), GABAA receptors, pharmacological inhibition, sensorimotor gating, muscimol

## Abstract

The deep and intermediate layers of the superior colliculus (DLSC) respond to visual, auditory, and tactile inputs and act as a multimodal sensory association area. In turn, activity in the DLSC can drive orienting and avoidance responses—such as saccades and head and body movements—across species, including in rats, cats, and non-human primates. As shown in rodents, DLSC also plays a role in regulating pre-pulse inhibition (PPI) of the acoustic startle response (ASR), a form of sensorimotor gating. DLSC lesions attenuate PPI and electrical stimulation of DLSC inhibits the startle response. While the circuitry mediating PPI is well-characterized in rodents, less is known about PPI regulation in primates. Two recent studies from our labs reported a species difference in the effects of pharmacological inhibition of the basolateral amygdala and substantia nigra pars reticulata (SNpr) on PPI between rats and macaques: in rats, inhibition of these structures *decreased* PPI, while in macaques, it *increased* PPI. Given that the SNpr sends direct inhibitory projections to DLSC, we next sought to determine if this species difference was similarly evident at the level of DLSC. Here, we transiently inactivated DLSC in four rhesus macaques by focal microinfusion of the GABA_A_ receptor agonist muscimol. Similar to findings reported in rodents, we observed that bilateral inhibition of the DLSC in macaques significantly disrupted PPI. The impairment was specific to the PPI as the ASR itself was not affected. These results indicate that our previously reported species divergence at the level of the SNpr *is not* due to downstream differences at the level of the DLSC. Species differences at the level of the SNpr and basolateral amygdala emphasize the importance of studying the underlying circuitry in non-human primates, as impairment in PPI has been reported in several disorders in humans, including schizophrenia, autism, and PTSD.

## Introduction

Sensorimotor gating—the ability to shift attentional resources to salient stimuli—is often operationalized and studied by assessing pre-pulse inhibition (PPI) of the acoustic startle response (ASR). In mammals the ASR is a rapid, reflexive contraction of skeletal, facial, and body muscles in response to a sudden, intense auditory stimulus (Davis et al., 1982; Koch and Schnitzler, 1997) and is conserved across many species, including rats, mice, guinea pigs, cats, dolphins, non-human primates, and humans (Saitoh et al., 1987; Wu et al., 1990; Geyer, 1999; Dawson et al., 2008; Dehmel et al., 2012; Saletti et al., 2014; Götz et al., 2020). PPI refers to the attenuation of the ASR when a subject is exposed to a low-intensity auditory “prepulse” prior to a startle-inducing auditory “pulse” (Geyer et al., 1990). PPI has been reported to be disrupted in several neuropsychiatric disorders, including schizophrenia, obsessive compulsive disorder, and post-traumatic stress disorder (PTSD) (Grillon et al., 1996; Kohl et al., 2013).

In rodents, both permanent lesions and transient pharmacological manipulation of basal ganglia input nuclei (e.g., nucleus accumbens), relay nuclei (e.g., ventral pallidum), and output nuclei (e.g., the substantia nigra pars reticulata; SNpr) disrupt PPI (Wan and Swerdlow, 1993; Kodsi and Swerdlow, 1997; Kretschmer and Koch, 1998; Forcelli et al., 2012; Aguilar et al., 2018). In a parallel study employing transient pharmacological inhibition of the SNpr in both rodents and non-human primates (Aguilar et al., 2018), we recently reported a surprising divergence of effect—while infusion of the GABA_A_ receptor agonist muscimol into the SNpr *disrupts* PPI in rodents, we found that the same manipulation *potentiates* PPI in rhesus macaques, suggesting that the contribution of basal ganglia circuits to PPI may differ between the rodent and primate brain.

The superior colliculus (SC), a major downstream target of basal ganglia efferent output, has a well-established role in PPI in rodents. Lesions of the SC disrupt PPI (Fendt et al., 1994), while transient blockade of GABA_A_ receptors in the SC augments PPI (Fendt et al., 1994; Fendt, 1999). Moreover, focal blockade of glutamate receptors in the deep and intermediate layers of the SC (DLSC) impairs PPI (Ding et al., 2019) and focal microstimulation of the DLSC can act as a pre-pulse and suppress auditory-evoked startle (Li and Yeomans, 2000).

The DLSC receive multimodal sensory input (visual, auditory, tactile), and coordinate eye, head, and whole-body movements to stimuli. Stimulation of the DLSC can evoke both orienting (e.g., saccades) and escape-like responses in rodents (McHaffie and Stein, 1982; Dean et al., 1989; Comoli et al., 2012), cats (Stein et al., 1980), and non-human primates (Hikosaka and Wurtz, 1983, 1985; DesJardin et al., 2013). In rodents, approach and avoidance behaviors display a medio-lateral and rostro-caudal topography (Dean et al., 1989; Comoli et al., 2012). By contrast, in non-human primates we have previously reported that activation of both medial and lateral superior colliculus produces avoidance responses (DesJardin et al., 2013). Consistent with this role for the SC in defense, avoidance, and threat detection, recent neuroimaging studies have revealed increased activity in the SC in individuals with PTSD both in response to prolonged eye contact (Steuwe et al., 2014) and in response to subliminal presentation of threat-related stimuli (Terpou et al., 2019). This is particularly interesting given that PPI and acoustic startle have been reported to be disrupted in PTSD (Ornitz and Pynoos, 1989; Butler et al., 1990; Grillon et al., 1996; Morgan et al., 1996; Shalev et al., 2000; Pineles et al., 2016). Specifically, two studies from Vietnam War veterans with combat-related PTSD reported significantly increased startle response and disrupted PPI, respectively (Butler et al., 1990; Grillon et al., 1996). These effects extend beyond combat-related trauma and are not specific to adults, nor are they dependent on sex. One study in children with PTSD and a second in women with PTSD both found a significant loss of PPI (Ornitz and Pynoos, 1989; Pineles et al., 2016).

Given (1) the recent reports of midbrain (superior colliculus) involvement in PTSD in clinical populations, and (2) that the DLSC are a primary efferent target of the SNpr, which we have found to differ in function with respect to PPI in rodents and primates, we sought to determine if the previously described species difference in the effect of SNpr inhibition on PPI was similarly present at the level of the DLSC. To address this, we microinjected a GABA_A_ receptor agonist, muscimol, into the DLSC of four macaque monkeys and assessed acoustic PPI through measurement of the whole-body ASR.

## Materials and Methods

### Experimental Design

To evaluate the role of the DLSC in sensorimotor gating in rhesus macaques, we measured PPI of the whole-body ASR of monkeys seated in a primate chair. Four rhesus macaques were implanted with cranial microinfusion platforms and infused bilaterally with muscimol into the DLSC for transient, reversible inactivation of the target region or bilateral injection of saline as a control. Manipulations were performed on a within-subject basis on a randomized treatment schedule.

### Subjects

Four male, rhesus macaques (*Macaca mulatta*) were used in this study (NO, SL, RE, and OD). At the age of 2–3 years, they were procured from AlphaGenesis and transferred to Georgetown University, where all experimental procedures were conducted. Monkeys were pair-housed within two joined individual cages (size, 61 × 74 × 76 cm each). The monkeys were housed in a room with a regulated 12 h light/dark cycle and maintained on a primate lab diet (Purina Mills, catalog #5049), supplemented with fresh fruit and vegetables. Water was available *ad libitum* in the home cage.

Care and housing of the monkeys at the Georgetown University Division of Comparative Medicine met or exceeded the standards as stated in the Guide for Care and Use of Laboratory Animals [National Research Council (US) Committee for the Update of the Guide for the Care and Use of Laboratory Animals, 2011], Institute for Laboratory Animal Research recommendations, and AAALAC International accreditation standards. The study was conducted under a protocol approved by the Institutional Animal Care and Use Committee at Georgetown University.

The present experiments began after the animals were extensively socialized and behaviorally trained (including chair training), which continued until the age of about 4 years. At the time of testing, animals ranged from 4–6 years old and weighed between 5 and 9 kg. In addition to the experimental procedures described here, all subjects were trained on various cognitive tasks administered at the Wisconsin General Testing Apparatus; the tasks included the Hamilton Search task and a task that tested reactivity to emotionally salient stimuli (Elorette et al., 2020). As part of those experiments, some animals received drug infusions in the basolateral amygdala (NO, SL, RE), SNpr (NO, SL), parahippocampal cortex (OD, RE, SL), hippocampus (OD, RE, SL), periaqueductal gray (RE, SL), and pulvinar (SL). Infusions into the DLSC for PPI experiments occurred toward the conclusion of all other experimentation and overlapped most prominently with injections into the hippocampus and parahippocampal cortex. We do not anticipate that the prior or concurrent infusions influenced the data for this project, as all of our animals exhibited the expected pattern of increasing PPI as a function of pre-pulse intensity after saline/control infusions. Disruptions to PPI were only observed following infusion of muscimol to the DLSC.

### Implantation of Drug Infusion Platform and Site Verification

**Figure 1:**
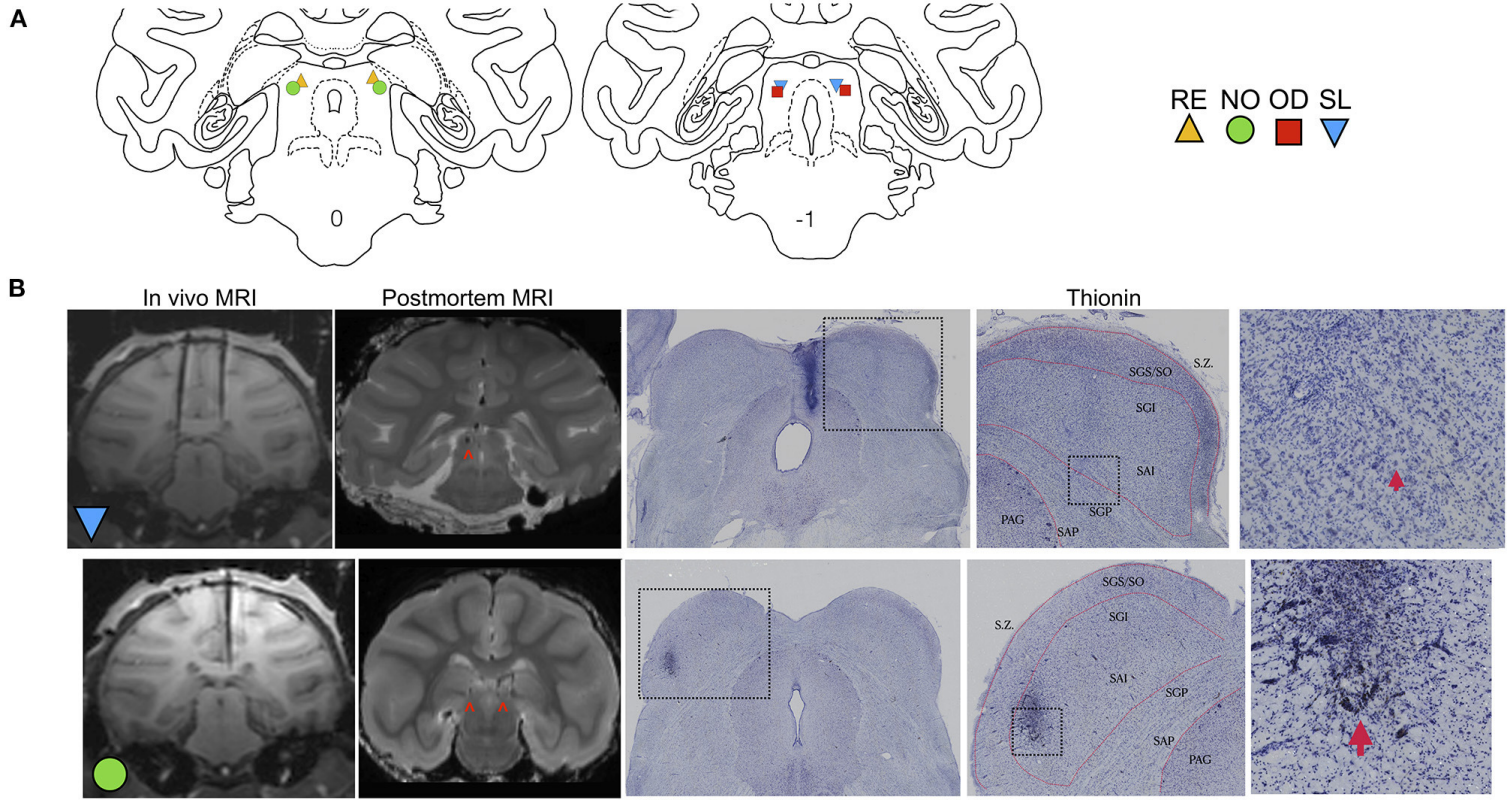
Localization of DLSC infusion sites. **(A)** Coronal sections of a macaque brain atlas showing anterior DLSC (0.0 mm) and posterior DLSC (−1.0 mm). Symbols indicate locations of injection sites for each animal (SL, blue triangle; OD, red square; RE, yellow triangle; NO, green circle), determined from *in vivo* MRI (all animals) and confirmed by post-mortem MRI and histology (two animals). **(B)** *In vivo* MRI used for site localization, post-mortem MRI for site confirmation, and histology for site confirmation. In the *in vivo* images, tungsten microelectrodes can be seen dorsal to the DLSC in SL and NO. In the both post-mortem MRI images and histological images, arrows point to the cannula tracts resulting from microinfusion of muscimol or saline into the DLSC. Boxes outline magnified images in subsequent panels. Layers of the SC are outlined in red. SZ, stratum zonale; SGS/SO, stratum griseum superficiale/stratum opticum (superifical layers); SGI, stratum griseum intermedium; SAI, stratum album intermedium; SGP, stratum griseum profundum; SAP, stratum album profundum; PAG, periaqueductal gray. As shown, there is minimal tissue damage to the SC, and injections fell within the deep and intermediate (SGI/SAI/SGP/SAP) layers.

The monkeys were implanted with a stereotaxically positioned chronic infusion platform, which enabled us to target specific sites within the DLSC based on the coordinates assessed by structural magnetic resonance imaging (MRI) scans. For the pre- and post-operative MRI and the surgery, we followed procedures as described in detail in our previous studies (Forcelli et al., 2016, 2017a,b; Wellman et al., 2016; Aguilar et al., 2018). Briefly, the infusion platform was implanted under anesthesia and aseptic conditions (Wellman et al., 2005; Forcelli et al., 2016), followed by a post-operative regimen of analgesics and antibiotics determined in consultation with the facility veterinarian. Postoperatively, each monkey received at least one T1-weighted structural MRI scan (0.75 × 0.75 mm in-plane resolution, 1 mm slice thickness) intended to obtain coordinates for infusions in the DLSC. Tungsten micro-electrodes (FHC; Bowdoin, ME), which were visible on the scan, were used to determine the precise coordinates as described previously (Holmes et al., 2012). Figure 1B shows the placement of a tungsten microelectrode at the dorsal border of the SC in two subjects. A figure of the infusion platform and telescoping cannula can be found in Wellman et al. (2005).

### Intracerebral Drug Infusions

To transiently inactivate the DLSC (for infusion sites, see Figure 1A), 9 nmol of the GABA_A_ receptor agonist muscimol (Sigma-Aldrich) in a volume of 1 ul (9 mM) was infused bilaterally at rate of 0.2 ul/min under aseptic conditions as previously described (Forcelli et al., 2017a). In our previously published studies, our lab found that injection of 1 μl of the contrast agent gadolinium (5 mM in saline) resulted in an area hypersignal of ~3 mm in diameter (DesJardin et al., 2013; Forcelli et al., 2014), suggesting a sphere of drug spread of ~3 mm an hour after infusion.

The entire infusion procedure lasted 10–15 min, and animals began the PPI procedure ~5–10 min after the microinfusion was completed. At least 48 h elapsed between drug treatments in an individual subject. Control infusions consisted of either microinjection of an equivalent volume of sterile saline or a “sham” infusion. For sham infusions, all procedures were followed, but no cannula was lowered into place; sham infusions were included to minimize the number of brain penetrations.

Across subjects, we found that sham infusions and saline infusions produced near-identical results. We tested for a difference between sham and saline infusions using a mixed-effects model with pre-pulse intensity and treatment (sham or saline) as fixed factors and monkey and test session as random effects. We found neither a main effect of treatment (*F*_1, 14.36_ = 0.489, *P* = 0.496) nor a treatment-by-pre-pulse intensity interaction (*F*_2, 34_ = 0.036, *P* = 0.964), and thus collapsed across these conditions for subsequent analyses. Sham infusions were performed in all animals; saline infusions were performed in RE, OD, and SL.

The number of infusions per animal ranged from 1 to 5 for muscimol and 2 to 6 for control. Variability in infusion number is due to loss of the cranial implant, as these were typically the last studies planned for each animal. The number of infusions per animal is shown in Table 1. In addition to the listed infusions, we dropped one session control and one muscimol session from Animal OD, and one session from Animal RE as they were outliers on a within-subject basis (ROUT test, *Q* = 0.1%).

**Table 1:** Number of injections performed in each subject.

**Monkey**	**SL 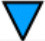 **	**NO 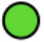 **	**RE 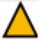 **	**OD 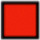 **
# of muscimol injections	5	1	4	3
# of Control injections	5 (1)	2	6 (2)	6 (2)

**Figure 2:**
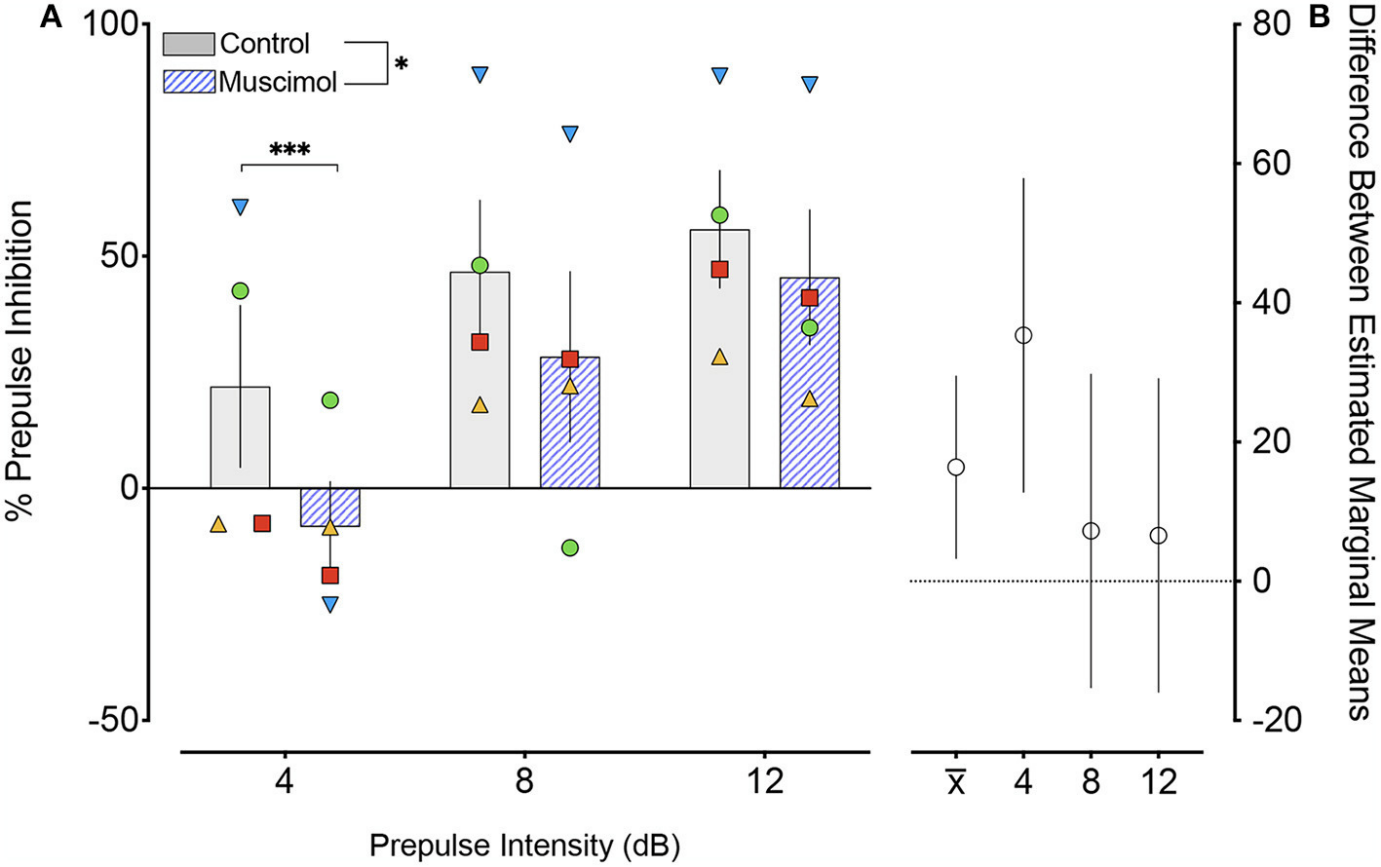
Muscimol infusion into the DLSC impairs PPI. **(A)** % PPI as a function of pre-pulse intensity (dB). Data show a main effect of drug treatment (*P < 0.05), apparent in the decreased % PPI following muscimol infusion. Pairwise comparisons revealed a significant decrease only with pre-pulse intensity of 4 dB (***P=0.002). The individual values for each animal are plotted as symbols (SL, blue triangle; OD, red square; RE, yellow triangle; NO, green circle). **(B)** Differences (and 95% confidence intervals) in estimated marginal means for the main effect of treatment ($\bar{\text{x}}$) and each pre-pulse intensity.

**Figure 3:**
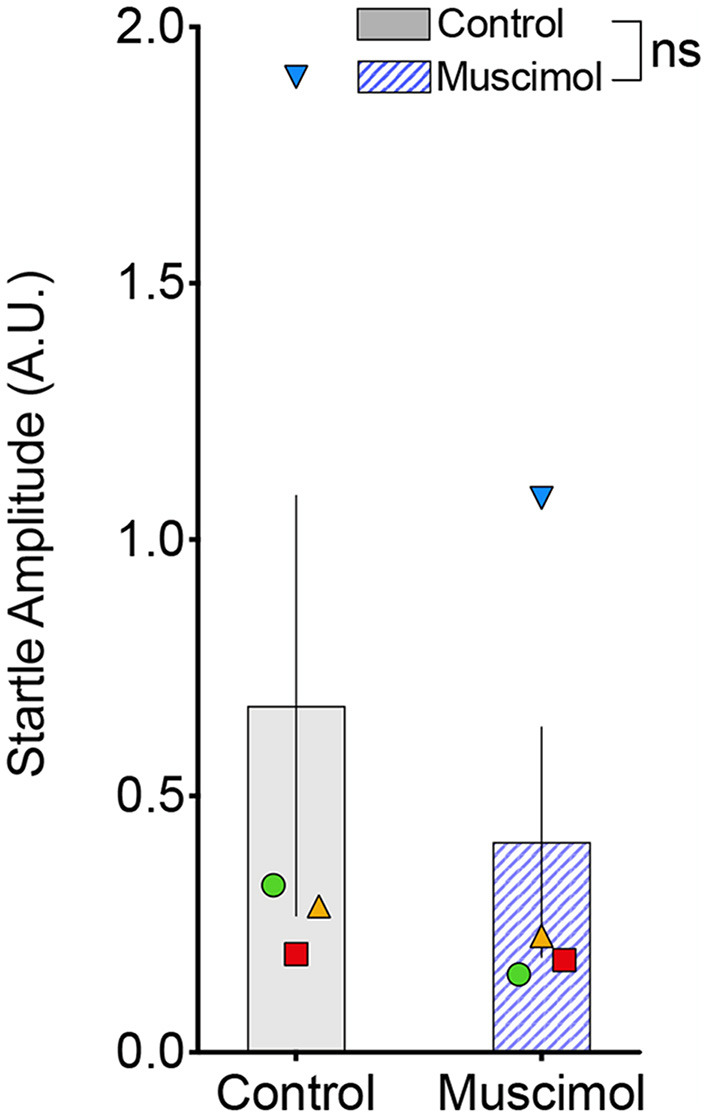
Muscimol infusion into the DLSC does not alter startle amplitude on pulse-alone trials. Each symbol indicates the average startle amplitude for each subject (SL, blue triangle; OD, red square; RE, yellow triangle; NO, green circle) following control or muscimol infusion. Average startle amplitude did not differ between treatment conditions.

*Number in parentheses indicate the number of saline infusions out of the total number of control injections per subject. Symbols correspond to those in Figures 1–3*.

### PPI Task Set-Up

PPI testing was conducted using an apparatus modified from that described by Winslow et al., 2002 and was conducted essentially as we have previously described (Aguilar et al., 2018). Tests were conducted in a behavior room located next to the home cage room, in a sound attenuated chamber containing a primate chair (Crist Instruments Co.) attached to a platform sitting on a load cell. The chamber (60 × 114 × 80 cm) also contained a speaker (25 cm above the head) for administration of noise stimuli. The primates' whole-body startle movements were transmitted via a 50 kg load cell (Sentran LLC; YG6-B) located between the chamber floor and the primate chair platform. The load cell was connected to an amplifier which transmitted a signal to a Windows XP computer running the Startle Response software (Med Associates). Prior to experimentation, we calibrated the amplifier using a 10 kg weight and maintained this calibration setting across all animals. All animals weighed between 6 and 9 kg, so this single calibration was sufficient.

Animals were habituated to the apparatus during a training period of about 3 weeks. During the first week, animals were placed in the PPI chamber daily for sessions increasing by 5 min each day, from 5 to 25 min, with the door open and while receiving continuous positive reinforcement from the experimenter (e.g., grapes). During the second week, animals were placed in the chamber daily for the same increasing session durations. However, during this phase, the door was closed and animals were exposed to white noise (70 dB). Animals were positively reinforced before and after door closure during this phase. During the third week, short (20 min) baseline sessions were run in which the animals were exposed to startling stimuli for the first time.

### PPI Protocol

Each 50 min session consisted of a 3-min acclimation period with background noise (70 dB), 6 blocks of 3 randomized startling stimuli (90, 105, 110 dB; 40 ms pulse for OD, RE, and SL; 90, 100, 105 for NO, who was tested on an older version of the protocol), 15 blocks of 4 randomized trials containing pulse-alone (105 dB; 40 ms) and prepulse-pulse (pre-pulses: 4, 8, and 12 dB above background noise; 20 ms) trials, and 10 blocks of 3 randomized startling stimuli (90, 105, 110 dB; 40 ms pulse). Pulse alone trials across the whole session were used as a control for maintenance of startle amplitude, and blocks containing startling stimuli at the beginning and end were used to calculate habituation to startle.

During the prepulse-pulse trials an inter-stimulus interval (onset to onset) of 50 ms was used based on our prior study (Aguilar et al., 2018). The inter-trial interval ranged from 15–30 s, randomly selected for each trial. Startle amplitude was defined as the peak load cell output voltage over a 175-ms period beginning at the onset of the pulse stimulus.

### Post-mortem MRI and Histology

SL, NO, and OD were perfused as previously described (Elorette et al., 2018). For *post-mortem* MRI analysis, the brains of three animals (SL, NO, and OD) were examined at high field strength (7 Tesla) on a Brucker Biospin Magnet using a Turbo-RARE pulse sequence, as previously described (Forcelli et al., 2016). Following MR imaging, brains were processed for localization of infusion sites, as we have previously described (Wellman et al., 2005; Gale et al., 2012; Forcelli et al., 2014). Representative photomicrographs and MR images are presented in Figure 1B.

### Data Pre-processing

On a session-by-session basis for each monkey, we performed automatic outlier removal using the ROUT (robust regression and outlier removal) test (*Q* = 10%) in GraphPad Prism 8. We have found that the within-subject, within-session variability of startle responses is often larger in macaques than in comparable studies in rodents, and thus removed high amplitude outliers which reflect motion in the chair unrelated to the startle response. We select a permissive removal criterion to remove all large amplitude, artifactual responses. The mean number of dropped trials for control sessions was 7.6, the mean number of dropped trials for muscimol sessions was 8.4, typically 1 to 3 trials were excluded for each trial type (i.e., pulse alone and each pre-pulse intensity). By dividing each session into thirds and calculating the number of trials excluded as outliers for each block within each session, we found that average number of dropped trials per session did not differ as a function of block (*F*_2,6_ = 0.81, *p* = 0.49), treatment (*F*_1,3_ = 0.51, *p* = 0.53), nor did we detect a block-by-treatment interaction (F_2,6_ = 0.87, *p* = 0.46). Thus we concluded that (1) our treatment did not influence the number of dropped trials and (2) habituation effects were unlikely to have influenced the rate of dropped trials.

Following artifact/outlier removal, we calculated PPI. PPI was defined as [1-(startle amplitude on pre-pulse trials/startle amplitude on pulse alone trials)] × 100 as previously described (Winslow et al., 2002; Davis et al., 2008). Startle habituation was calculated as: [1-(mean response on post-test trials/mean response on pre-test trials)]*100 on a session-by-session basis.

### Statistical Analysis

We used the MIXED procedure in SPSS (Version 25) for data analysis with Restricted Maximum Likelihood estimation and the Satterthwaite approximation for degrees of freedom calculations. GraphPad Prism 8 was used for figure preparation.

ASR on pulse-alone trials was analyzed using a mixed-effects model with treatment as a fixed effect and monkey and session as random effects. PPI data were analyzed using a multi-level marginal model in SPSS, with treatment and pre-pulse intensity as fixed effect factors. Note that we also analyzed these data using a mixed effects model with treatment and pre-pulse intensity as fixed effects and monkey and session as random factors. This produced a model with poorer fit (based on both Akaike information criterion and Bayesian information criterion metrics) than the marginal model, although the fixed effects and pairwise comparisons of the estimated marginal means produced similar results. For this model, we generated estimated marginal means and present the difference in estimated marginal means, along with 95% confidence intervals of the differences as a visual estimation of effect size. As deficits in PPI are often overcome at higher pre-pulse intensities, we planned *a priori* to compare PPI as a function of treatment within each pre-pulse intensity. The ASR curve on the pre-test and post-test trials was analyzed using a mixed-effects model with treatment, block (beginning of session vs. end of session), and noise amplitude (90, 105, 110 dB) as fixed factors and monkey and session as random effects. Startle habituation was analyzed using a mixed-effects model with treatment and noise amplitude as fixed effects and monkey and session as random effects.

## Results

Infusion site verification is shown in Figure 1. The position of electrodes from the *in vivo* scans closely correspond to the localization of the cannulae tracks from post-mortem MRI scans and histological reconstruction. Histological analysis confirmed localization of infusion cannulae to the DLSC, and not the superficial layers of superior colliculus. Representative MR images and photomicrographs are shown in Figure 1B. Sites were further confirmed using behavior from a parallel set of experiments with these subjects: unilateral activation of the DLSC at the same site using bicuculine methiodide (2.5 nmol) resulted in contralateral saccades and defensive vocalizations in SL, NO, and RE, consistent with prior reports (DesJardin et al., 2013).

The effects of muscimol infusion into the DLSC on PPI are shown in Figure 2. As expected, we found a main effect of pre-pulse intensity (*F*_2,87.2_ = 24.229, *p* = 0.000000004), with increasing pre-pulse intensity associated with higher levels of PPI in both control (linear regression, *R*^2^ = 0.1704, *p* = 0.0014) and muscimol-infused conditions (linear regression, *R*^2^ = 0.2752, *p*=0.0002). We also found a main effect of treatment (*F*_1,87.4_ = 6.198, *p* = 0.014), but no pre-pulse intensity-by-treatment interaction (*F*_2,87.2_ = 2.113, *p* = 0.127). The main effect of treatment (indicated by x) is evident in difference in estimated marginal means plot shown in Figure 2. *A priori*, we planned to compare drug within each level of pre-pulse intensity. The treatment effect was driven by a significant reduction in PPI when pre-pulse intensity was 4 dB above background (*p* = 0.002, Sidak corrected). No significant differences were found for pre-pulse intensities of 8 (*p* = 0.525, Sidak corrected) or 12 (*p* = 0.563, Sidak corrected) dB above background. The magnitude of the drug effect for each pre-pulse intensity is shown in the plot of differences in estimated marginal means in Figure 2.

A change in magnitude of the ASR (i.e., the response to pulse-alone trials without a prepulse) may contribute to or mask changes in PPI (Sandner and Canal, 2007; Shoji and Miyakawa, 2018). In rodents, lesions (Groves et al., 1974; Fendt et al., 1994) and pharmacological activation (Fendt, 1999) of the DLSC have been reported to have no effect on baseline startle. However, one report found *enhanced* baseline startle response after DLSC lesions in rodents (Tischler and Davis, 1983). Consistent with the majority of the existing literature, we found no effect of muscimol infusion in SC on baseline startle amplitude (*F*_1,7.4_ = 1.053, *p* = 0.337, mixed effects model, Figure 3). We did, however, note that the average startle amplitude for each of the four animals was numerically decreased after muscimol infusion.

**Figure 4:**
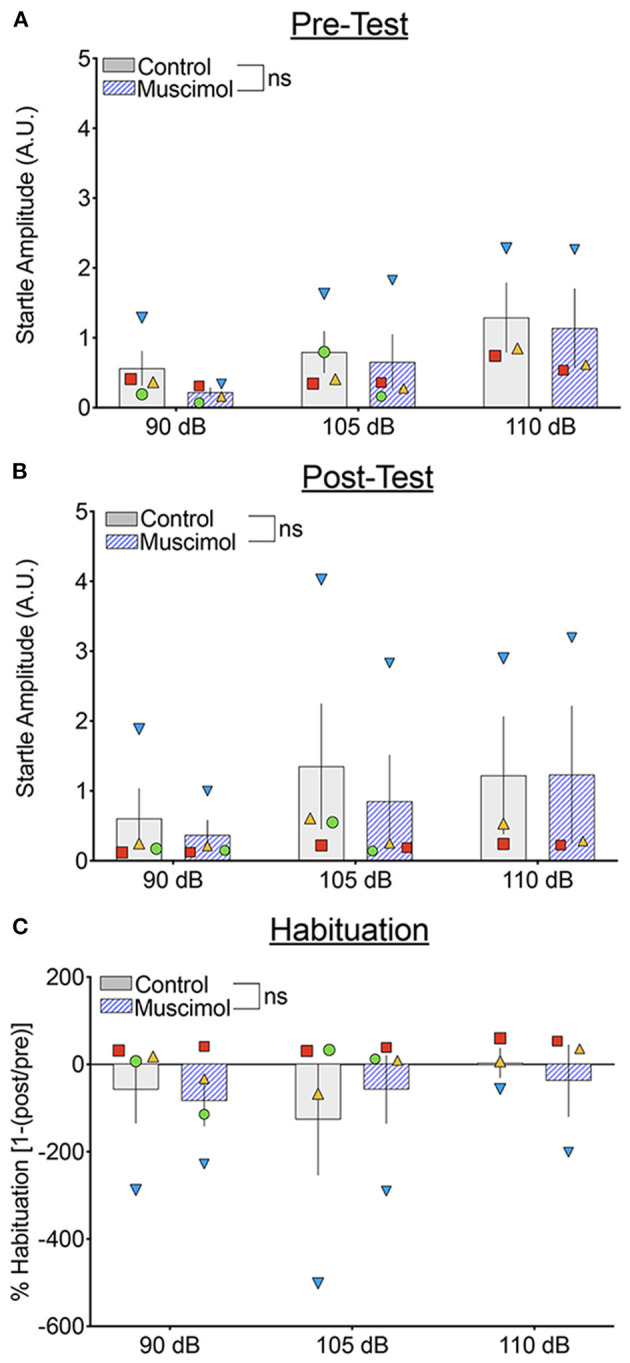
Muscimol infusion into the DLSC does not alter baseline startle amplitude or startle habituation during pre- or post-test trial blocks. Each symbol indicates the average startle amplitude for each subject (SL, blue triangle; OD, red square; RE, yellow triangle; NO, green circle) following control or muscimol infusion. **(A)** Startle amplitude as a function of drug treatment and noise intensity during the pre-test trial block. **(B)** Startle amplitude as a function of drug treatment and noise intensity during the post-test trial block. **(C)** Percent habituation of the startle response comparing the pre and post-test trial blocks.

We next compared the startle response curves as a function of noise amplitude during the pre- and post-test blocks (Figures 4A,B, respectively). We found a significant main effect of noise amplitude (*F*_1,41.071_ = 15.468, *p* = 0.000316), a borderline-significant effect of trial block (pre-test vs. post-test, *F*_1,93.322_ = 3.667, *p* = 0.059), but no significant main effect of treatment (*F*_1,39.241_ = 1.41, *p* = 0.242). The two-way interaction between treatment and noise amplitude was not significant (*F*_1,41.071_ = 0.688, *p* = 0.412), nor was the interaction between treatment and trial block (*F*_1,93.322_ = 0.01, *p* = 0.920). The three-way interaction between treatment, trial block, and noise amplitude also did not reach the level of statistical significance (*F*_2,100.296_ = 1.150, *p* = 0.321). Given the trend toward a main effect of trial block, we also examined habituation of startle Figure 4C). We found no significant effect of treatment (*F*_1,33.224_ = 0.006, *p* = 0.937), noise amplitude (*F*_2,56.374_ = 1.667, *p* = 0.198), or a treatment-by-noise amplitude interaction (*F*_2,56.676_ = 0.575, *p* = 0.566).

## Discussion

Here we report that inactivation of the DLSC by microinjection of the GABA_A_ agonist, muscimol, significantly disrupts PPI, as measured through a whole-body startle response paradigm, in rhesus macaques without altering startle amplitude. These data add to a small but growing literature examining the pharmacology, physiology, and neural circuitry controlling PPI in non-human primates.

The only other non-human primate study to examine the superior colliculus in the context of PPI—conducted by Saletti et al. in two capuchin monkeys—noted reduced PPI in both lesioned animals (Saletti et al., 2014). Our present findings, as well as the findings from Saletti et al. are consistent with the impairments in PPI observed following SC lesions in rats (Fendt et al., 1994). More generally, our findings also support the well-established role of the DLSC as a sensorimotor integrator. The SC receives visual, auditory, and somatosensory information and influences motor outputs (May, 2006). In rodents, two categories of motor responses are observed upon stimulation of the SC: one class is characterized by orienting responses, such as tracking or pursuit, and the second is characterized by defensive movements, such as avoidance or flight responses (Dean et al., 1989). These behaviors are topographically organized within the SC, with the medial DLSC playing an important role in avoidance behavior and the lateral DLSC playing a role in orienting and approach (Sahibzada et al., 1986; Dean et al., 1989). These fundamental avoidance and approach motor programs are mediated by uncrossed and crossed projections through the brainstem, respectively (Dean et al., 1989). In primates, disinhibition of the DLSC with bicuculline methiodide results in universal defensive responses, regardless of medial vs. lateral activation (DesJardin et al., 2013). While the small number of subjects in the present study precludes a definitive assessment of medial-lateral topography for effects on PPI, we note that both our most medial case (SL) and our most lateral case (NO) showed deficits in PPI after muscimol infusion. A lack of topography in the monkey would be consistent with our prior report on defense-like behaviors (DesJardin et al., 2013). Thus, despite the differences in topography and behavior observed following activation of the rodent SC and the nonhuman primate SC, the role of the SC appears conserved in the context of PPI.

We observed no significant effect of DLSC inactivation on startle habituation during the testing sessions, consistent with a report in rodents showing a lack of effect of superior colliculus lesions on acoustic startle or habituation to startle (Groves et al., 1974). However, this similarity must be interpreted with caution as we did not observe robust startle habituation under baseline conditions. While startle habituation has been routinely reported in mouse and rat studies (Groves et al., 1974; Anisman et al., 2000; Geyer and Swerdlow, 2001; Sandner and Canal, 2007; Partridge et al., 2016), neither we (present study) nor others (Parr et al., 2002) found significant startle habituation in the macaques. This is notable, as our PPI paradigm used a larger number of habituation trials than are typically used in rodents. Similar to our findings, Saletti et al. found no evidence for startle habituation in capuchins. However, habituation to startle-inducing broadband noise has previously been reported in squirrel monkeys (Parker et al., 2011). Moreover, in a fear-potentiated startle paradigm in macaques, within-session habituation was observed between the first trial block and subsequent trial blocks (Winslow et al., 2007), although this effect was modest in the absence of a conditioned stimulus. Finally, Schneider et al. reported significant habituation to startling stimuli across the first four trials of an acoustic startle test in macaques (Schneider et al., 2013). Unfortunately, the remaining studies of acoustic startle (Javitt and Lindsley, 2001; Sánchez et al., 2005; Henry et al., 2009), fear potentiated startle (Antoniadis et al., 2007, 2009; Kazama et al., 2012, 2013), or PPI (Javitt and Lindsley, 2001; Linn and Javitt, 2001; Linn et al., 2003; Nelson et al., 2009; Morris et al., 2010) in non-human primates did not report data regarding startle habituation. Accordingly, there is not a clear consensus on the degree to which habituation of the whole-body ASR is a robust effect in non-human primates.

PPI is disrupted in several neuropsychiatric disorders, including schizophrenia, obsessive compulsive disorder, and PTSD (Grillon et al., 1996; Kohl et al., 2013). Understanding the circuit organization of these disorders critically relies on homology between models and humans, and non-human primates offer both behavioral and circuit homology that makes them ideally suited for this purpose. Particularly in light of our previous findings of species differences in PPI circuitry, we suggest that further evaluation of the neural circuitry controlling sensorimotor gating in the primate will be informative and of translational value for human neuropsychiatric conditions. Compared to the more extensive literature on acoustic and fear potentiated startle, there have been far fewer studies investigating PPI in primate models as compared to rodents, and fewer still examining circuitry underlying PPI in primates (a total of four studies, including the present). In addition to the study by Saletti et al. described above in capuchin monkeys, the only other circuit-based studies have been from our labs and have focused on the SNpr and the basolateral amygdala. In both cases, findings differed strikingly from those observed in rodents. We found that inactivation of the basolateral amygdala facilitated PPI in macaques (Elorette et al., 2020), a pattern opposite to that observed in rodents, where both permanent damage or transient inhibition of the basolateral amygdala disrupts PPI (Wan and Swerdlow, 1996; Fendt et al., 2000; Forcelli et al., 2012). Similarly, we reported that inactivation of the SNpr in macaques facilitated PPI, whereas in rats it impaired PPI (Aguilar et al., 2018). Following SNpr inactivation, we observed behaviors that we and others have previously shown to be mediated by disinhibition of the SC (DesJardin et al., 2013; Aguilar et al., 2018). We thus hypothesized that this species difference was due to our selective targeting of nigral neurons projecting to the DLSC; these neurons do not collateralize in the primate, but do collateralize and project to other regions (e.g., the pedunculopontine nucleus) in rodents (Yasui et al., 1995; Mailly et al., 2003; Cebrián et al., 2005).

While the effects of inhibition of the DLSC appear consistent across species, we did not study the effect of disinhibition of the DLSC on PPI in the present study. Given that disinhibition of the DLSC can produce motor confounds (defensive responses, postural asymmetries), we were concerned that interpretation of whole-body startle responses would be ambiguous in primates. However, it is well-established that inhibition of the SNpr (as in our prior study) produces a *disinhibitory* (activating) effect on the DLSC. In rodents, intracollicular microinjection of picrotoxin, a GABA chloride channel blocker, enhances PPI without altering ASR amplitude (Fendt, 1999). Similarly, electrical stimulation of the DLSC inhibits the startle reflex in rodents (Li and Yeomans, 2000). Given that our present findings with inhibition of the DLSC are consistent with those reported in rodents—and were expected based on the functional relationship between the SNpr and DLSC—it is likely that nigral projections to the pedunculopontine nucleus in the rodent may explain the previously reported species difference (Aguilar et al., 2018). In rodents, both increased or decreased activity of the PPN results in PPI deficits, suggesting that the PPN is highly sensitive to alterations in neurotransmission in the context of PPI (Koch et al., 1993; Swerdlow and Geyer, 1993; Koch and Schnitzler, 1997; Takahashi et al., 2007). In primates, the role of the PPN in PPI is completely unexplored.

Here, our data suggest that activity within the DLSC is necessary for normal PPI in macaques, as has been reported in rodents. Our findings differ from reports from our labs following inhibition of the SNpr or basolateral amygdala, which showed opposing results in macaques (increased PPI) and rats (decreased PPI). As so little is known regarding the functional organization of PPI circuitry in the primate, both the similarities and the differences highlight the importance of better understanding of these circuits. Given the diverse neuropsychiatric and neurological conditions in humans that are associated with disrupted PPI, a more detailed understanding of the circuitry regulating this phenomenon in the primate brain is clearly warranted.

## Data Availability Statement

The raw data supporting the conclusions of this article will be made available by the authors, without undue reservation.

## Ethics Statement

The animal study was reviewed and approved by Georgetown University Animal Care and Use Committee.

## Author Contributions

BA, LM, and PF: conceptualization and design. HW, BA, LM, and PF: experimentation, wrote and edited manuscript. HW, BA, and PF: formal analysis. LM and PF: supervised research. All authors contributed to the article and approved the submitted version.

## Conflict of Interest

The authors declare that the research was conducted in the absence of any commercial or financial relationships that could be construed as a potential conflict of interest.
